# Selecting Sample Preparation Workflows for Mass Spectrometry-Based Proteomic and Phosphoproteomic Analysis of Patient Samples with Acute Myeloid Leukemia

**DOI:** 10.3390/proteomes4030024

**Published:** 2016-08-22

**Authors:** Maria Hernandez-Valladares, Elise Aasebø, Frode Selheim, Frode S. Berven, Øystein Bruserud

**Affiliations:** 1 Department of Biomedicine, Faculty of Medicine and Dentistry, University of Bergen, Jonas Lies vei 91, N-5009 Bergen, Norway; Elise.Aasebo@uib.no (E.A.); Frode.Selheim@uib.no (F.S.); Frode.Berven@uib.no (F.S.B.); 2 Department of Clinical Science, Faculty of Medicine and Dentistry, University of Bergen, Jonas Lies vei 91, N-5009 Bergen, Norway; Oystein.Bruserud@uib.no

**Keywords:** acute myeloid leukemia, proteomics, phosphoproteomics, sample preparation, FASP, SILAC, mass spectrometry, IMAC, StageTip, biomarker

## Abstract

Global mass spectrometry (MS)-based proteomic and phosphoproteomic studies of acute myeloid leukemia (AML) biomarkers represent a powerful strategy to identify and confirm proteins and their phosphorylated modifications that could be applied in diagnosis and prognosis, as a support for individual treatment regimens and selection of patients for bone marrow transplant. MS-based studies require optimal and reproducible workflows that allow a satisfactory coverage of the proteome and its modifications. Preparation of samples for global MS analysis is a crucial step and it usually requires method testing, tuning and optimization. Different proteomic workflows that have been used to prepare AML patient samples for global MS analysis usually include a standard protein in-solution digestion procedure with a urea-based lysis buffer. The enrichment of phosphopeptides from AML patient samples has previously been carried out either with immobilized metal affinity chromatography (IMAC) or metal oxide affinity chromatography (MOAC). We have recently tested several methods of sample preparation for MS analysis of the AML proteome and phosphoproteome and introduced filter-aided sample preparation (FASP) as a superior methodology for the sensitive and reproducible generation of peptides from patient samples. FASP-prepared peptides can be further fractionated or IMAC-enriched for proteome or phosphoproteome analyses. Herein, we will review both in-solution and FASP-based sample preparation workflows and encourage the use of the latter for the highest protein and phosphorylation coverage and reproducibility.

## 1. Introduction

Acute myeloid leukemia (AML) is a hematopoietic disease distinguished by the rapid growth of abnormally differentiated myeloid cells in the bone marrow and peripheral blood [1,2]. The proliferation of the leukemic blasts interferes with the normal hematological system and it is frequently seen as a reduced production of healthy blood cells. AML is the most lethal type of acute leukemia as it is frequently associated with relapse and drug resistance. The average five-year survival percentage is only 26.6% [3], but the predicted survival prognosis differs based on risk factors, such as molecular abnormalities and age [1]. AML is divided into subtypes M0 through M7 (based on the type of cell from which the leukemia develops and how mature the cells are) in the French-American-British (FAB) classification of AML [4]. In the World Health Organization (WHO) system, AML is subclassified according to cellular morphology, hematopoietic lineage as well as known translocations and mutations [5]. These factors contribute to different therapy responses in patients, and render the prognostication and choice of treatments difficult [1]. The causes of AML are mostly unknown although recent studies have suggested an inherited predisposition of AML by germline mutations in AML-associated genes, like Runt-related transcription factor 1 (RUNX1) and CCAAT/enhancer binding protein alpha (CEBPA) [6,7].

Great effort has been put into understanding the biological complexity of AML, aimed at improving the prognostication and therapy. Specially, mass spectrometry (MS)-based proteomic studies on AML patient samples have been increasingly published in the past years. Most of them have been recently reviewed by another research group [8] and us [9]. Early MS-based proteomic studies on AML, which have mostly been carried out with two-dimensional electrophoresis (2DE)-based approaches combined with matrix-assisted laser desorption/ionization time-of-flight (MALDI-TOF)- or liquid chromatography (LC)-MS, resulted in a low number of quantified proteins compared to shotgun proteomics. Even so, several potential AML diagnosis, prognosis and treatment-response biomarkers were described from such studies. Current proteomic methods carried out using highly sensitive and fast mass spectrometers allow the identification and quantification of several thousands of proteins and post-translational modifications (PTM) from a single patient sample. However, proteomic and phosphoproteomic workflows are not universal and they might work differently depending on the characteristics of the sample and the experience of the researcher. Published global MS proteome and phosphoproteome analysis of AML patient samples have used the standard one-dimensional electrophoresis (1DE), 2DE and the in-solution digestion method with urea in the lysis buffer to digest AML proteins prior to MS analysis or phosphorylation enrichment [10,11,12,13,14]. We have recently tested several methods of sample preparation for the MS analysis of the AML blasts that included in-solution digestion methods with urea and guanidinium hydrochloride as denaturant reagents in the lysis buffer and filter-aided sample preparation (FASP) [15,16]. Several methods of phosphorylation enrichment, immobilized metal affinity chromatography (IMAC) [17], metal oxide affinity chromatography (MOAC) [18] and sequential elution from IMAC (SIMAC) [19] were also included in our tests. Herein, we will briefly review the sample preparation procedures used in previous global MS AML studies and we will describe those used in our method testing. We will support the FASP procedure as a reproducible and high-proteome-coverage strategy to investigate AML samples.

## 2. Sample Preparation Workflows in Global MS Proteome Studies of AML

The first MS-based global proteomic study addressed the protein profiling of AML FAB subtypes (M1 and M2) and whether the sample collection method (bone marrow or peripheral blood sampling) might influence the 2DE-based identification of AML biomarkers [10]. The authors suspended 71 and 57 AML samples of peripheral blood and bone marrow, respectively, with an isoelectric focusing buffer containing 7 M urea, 2 M urea and 2% CHAPS, loaded from 500 to 800 µg proteins on different pH strips and digested with trypsin before being run on MALDI-TOF or electrospray ionization (ESI)-MS/MS as mass spectrometers (Bruker Daltonics, Billerica, MA, USA). This proteomic workflow identified 861 proteins.

The first MS-based phosphoproteomic study with peripheral blood samples from 14 AML patients (in addition to two AML cell lines) introduced KSEA, an untargeted kinase-substrate enrichment analysis, for the systematic profiling of kinase pathway activities [11]. AML samples were lysed with a buffer containing 8 M urea and several potent inhibitors of protein phosphatases such as sodium vanadate and okadaic acid. Protein digestion was performed with immobilized trypsin after reducing the urea concentration to 2 M. The peptides were desalted and enrichment of phosphorylated peptides was performed with TiO_2_ affinity beads (MOAC) as described by Montoya et al. [20]. Using 500 µg of AML protein lysate, 5792 phosphopeptides were identified from the 14-patient cohort on an LTQ-Orbitrap XL mass spectrometer (Thermo Fisher Scientific, Waltham, MA, USA). Although the phosphoproteomic workflow presented in this study comprised a few steps of low technical difficulty, the use of urea in the lysis buffer required the addition of expensive and toxic phosphatase inhibitors that need to be handled with care.

The first MS-based phosphoproteomic study with bone marrow samples investigated the response to treatment with quizartinib, a tyrosine kinase inhibitor, in a patient cohort with six responders and six non-responders [12]. The AML cells were lysed with a buffer containing 8 M urea, sodium vanadate (among several other protein phosphatase inhibitors) and commercially available phosphatase inhibitor cocktail and protease inhibitor cocktail tablets. The average protein amount from the patient samples was approximately 450 µg. Protein digestion was first performed with Lys-C followed by trypsin. The desalted peptides were fractionated by strong cation exchange (SCX). Twelve fractions were collected and desalted before phosphopeptide enrichment with IMAC. More than 13,236 phosphosites were identified with an LTQ-Orbitrap Velos (Thermo Fisher Scientific). This phosphoproteomic workflow used an expensive lysis buffer, two proteases and involved the use of a complex fractionation step that drastically increased the time and cost of the mass spectrometer usage. The cost and complexity of this sample preparation strategy might not be applicable when larger patient cohorts need to be analyzed.

Src homology 2 (SH2) domains are phosphotyrosine-binding modular structures found mainly in several signaling and adaptor proteins for intracellular signal transduction. Moreover, SH2 domains of Src family kinases are themselves tyrosine phosphorylated in some blood cancers including AML [13]. To study the binding properties of those tyrosine-phosphorylated SH2 domains to phosphotyrosine-containing polypeptides and proteins, peripheral blood mononuclear cells from 12 AML patients were collected and lysed with a buffer containing 9 M urea and sodium vanadate among several phosphate-containing molecules that served as phosphatase substrates. The trypsin digested samples were enriched for phosphotyrosine peptides using PhosphoScan reagents (Cell Signaling Technology, Danvers, MA, USA) and analyzed with a Q Exactive or Elite (Thermo Fisher Scientific) mass spectrometers [13]. Sixty phosphotyrosine peptides were identified with an adapted phospho-enrichment step using 2 mg of cell lysate [21].

A recent MS-based proteomic study of the secretome from apoptosis-resistant AML blasts was performed to characterize how the secreted microenvironment influences apoptosis regulation in neighboring cells. The authors used 1DE with polyacrylamide gels to resolve protein bands before in-gel digestion with trypsin [14]. The analysis of whole secretome from 11 (five with high anti-apoptosis index (AAI), six with low AAI) bone marrow samples of AML patients identified 1492 proteins whereas 4232 proteins were identified from extracellular vesicles prepared from the bone marrow samples of only two AML patients (one with high AAI, one with low AAI) from the first cohort using LTQ-FTMS and Q Exactive mass spectrometers (Thermo Fisher Scientific), respectively. For each sample, 60–80 µg of protein was loaded onto the precast gels using lithium dodecyl sulfate (LDS)–based reducing sample buffer (Invitrogen, Waltham, MA, USA). Although the 1DE approach was able to identify a satisfactory number of proteins using a cheap sample preparation method, this strategy might not be applicable when larger patient cohorts need to be analyzed.

## 3. Testing of Standard and Novel MS-Based Proteomic and Phosphoproteomic Workflows

**Figure 1:**
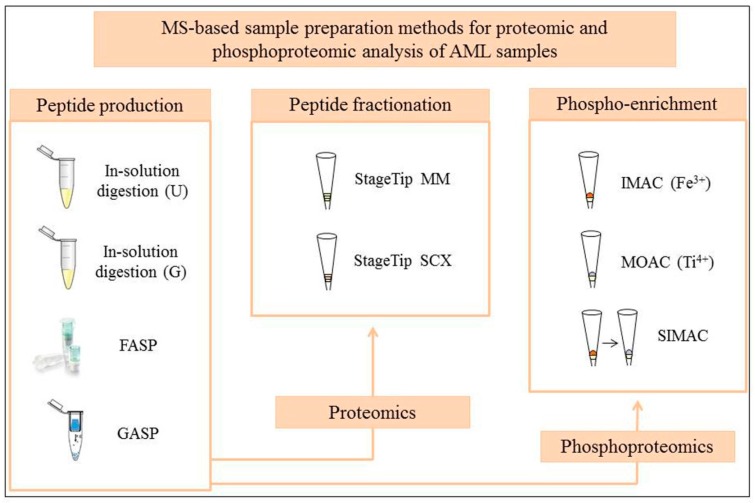
Proteomic and phosphoproteomic strategies tested on AML patient samples. The FASP protein filters and the GASP “reactor tube” illustrations were taken from manufacturer’s online resources and from the GASP publication [29] with permission from Wiley-VCH Verlag GmbH and Co. KGaA, respectively; U stands for urea, G for guanidinium hydrochloride, MM for mixed mode and SCX for strong cation exchange.

Besides the standard in-solution digestion strategy with urea in the lysis buffer, several methods to prepare samples for large MS-based proteomic and PTM studies have been developed in the past years (Figure 1). The spin filter approach, first introduced by Manza et al. [22], and the FASP approach [16] have become popular sample preparation methods in the proteomics community and they offer the use of a cheap sodium dodecyl sulfate (SDS)-based lysis buffer with no need to add costly protease and phosphatase inhibitors [16,22]. Conventionally, it is performed with one protease (trypsin) but the sequential use of two proteases (Lys-C and trypsin) has been shown to increase the proteome and phosphoproteome coverage of the sample [23,24,25]. With a StageTip format, a new in-solution approach named the in-StageTip method has been described to allow deep proteome coverage and high quantification accuracy [26]. In-StageTip can be performed with different lysis buffers that contain 8 M urea, 6 M guanidinium hydrochloride or 1% (*w*/*v*) sodium deoxycholate in addition to tris(2-carboxyethyl)phosphine (TCEP) and chloroacetamide (CAA) for simultaneous reduction and alkylation at high temperature. However, the use of the urea lysis buffer at temperatures higher than 40 °C is not recommended in order to prevent protein carbamylation. Optimized in-solution methods using trifluoroethanol as a protein denaturant [27] or guanidinium hydrochloride in the presence of TCEP and CAA [28] are attractive in-solution digestion alternatives that do not require additional protease and phosphatase inhibitors during cell lysis. Although 1DE is still of use for many MS-based proteomic studies (see [14] as an example in AML MS-based proteomics), an in-gel protocol that copolymerizes the entire protein sample with monomeric acrylamide and allows the depletion of denaturant agents used in the lysis buffer before proteolysis has been recently introduced as gel-aided sample preparation (GASP) [29]. The analogue acronym to FASP reflects similar working principles and workflows of both approaches.

In order to find out the optimal quantitative workflows for MS studies of AML patient samples, we have recently tested most of the sample preparation methods described above [15]. We used blast cells from peripheral blood of an AML patient for all the proteomic workflows while we used pooled samples from several AML patients for all the phospho-enrichment workflows. Although the tests were carried out with label-free and stable isotope labeling with amino acids in cell culture (SILAC)-labeled AML patient samples, we will only review herein our results from the SILAC-quantified samples. We used 20 µg of AML lysate and 20 µg of super-SILAC mix [30] for each proteomic workflow. We have also included two fractionation strategies using the StageTip format and the procedures with polystyrenedivinylbenzene reversed phase sulfonate (SDB-RPS, so-called mixed mode, MM) and SCX plugs described by Kulak et al. [26]. For enrichment of phosphopeptides, we tested IMAC, MOAC and SIMAC workflows [17,18,19] on the peptide samples produced from selected sample preparation strategies using 315–400 µg of AML lysate and the corresponding half amount of the super-SILAC mix.

Samples were analyzed on an Orbitrap Elite mass spectrometer coupled to an Ultimate 3000 Rapid Separation LC system (Thermo Fisher Scientific). The mass spectrometer was operated in the data-dependent acquisition (DDA) mode and ions were fragmented by low-energy collision-induced-dissociation (CID). Similar amounts of peptides and phosphopeptides were loaded. We kept the MS acquisition time constant or comparable for all the analyses. A detailed description of the LC-MS conditions can be found in the Supplemental Methods of our comparison study [15].

Our comparisons of the proteomic workflows revealed two important results (Table 1): (i) as expected, in-solution digestion approaches worked better with fractionation than with the use of one or two proteases; the in-solution digestion method (including fractionation) with guanidinium hydrochloride in the lysis buffer clearly outperformed the in-solution digestion method with the classical urea lysis buffer; (ii) without fractionation, the FASP approach produced the highest number of quantified protein groups when compared to the in-solution digestion methods; the FASP approach followed by the MM fractionation produced the highest number of quantified protein groups among all the workflows tested in the study. Between the in-solution with guanidinium hydrochloride and FASP, both followed by fractionation, we chose the FASP strategy with MM fractionation and with the lowest percentage of missed cleavages observed in the comparison study. This was an important factor to consider as a high percentage of missed cleavages can impair the protein match from MS data [31]. We did not include our quantitative proteomic results using the GASP procedure. We were not able to remove the SDS used in the lysis of AML patient samples, even after increasing the number of urea/acetonitrile washes as recommended [29]. This had a deleterious effect on the analytical column of our LC-MS system and provided a low number of quantified protein groups (data not shown).

The multi-steps of the FASP procedure together with increasing reports of filter failure [25,32] have discouraged the use of this strategy in the proteomics community despite its proven efficiency as in our comparison study. The use of faulty filters in the FASP protocol will cause the loss of most of the proteins in the sample as they are not retained on the filter membrane, yielding a poor peptide recovery after proteolytic digestion [32]. A reduced centrifugal speed has been suggested to avoid FASP failure [25]. However, an initial simple quality filter test with the urea FASP buffer worked best in our hands to differentiate working from failing protein filters [32].

Using the FASP procedure as our tool to produce peptides for phospho-enrichment, we highlighted the outstanding performance of the IMAC methodology with AML patient samples (Table 2) and its capability to enrich monophospho-, diphospho- and multiphospho-peptides. The use of two proteases did not remarkably increase the number of quantified localized phosphosites and the total number of the different phosphopeptides as suggested by others [23]. The double-digestion approach in the FASP protocol might be more suitable for samples of higher phosphorylation complexity than the one found in AML patient samples. Therefore, we chose the IMAC strategy for the enrichment of phosphopeptides prepared with the FASP/trypsin procedure on AML patient samples. As remarked in our comparison study [15], we found it difficult to explain the poor performance of the MOAC (and consequentially that of the SIMAC) phospho-enrichment, although the presence of acidic amino acids within the AML phosphopeptide sequences might explain the better specificity of the IMAC approach [33].

## 4. Discussion and Conclusions

Successful MS-based global proteomic and phosphoproteomic studies of AML patient samples require the use of optimal sample preparation methods to achieve a satisfactory proteome and phosphoproteome coverage. The choice of those methods is not arbitrary and it will depend on the nature and quantity of the samples as well as the researcher’s confidence and the availability of the necessary materials and equipment. These factors together with an increasing variety of sample preparation methods encourage testing studies to find optimal workflows that might cover the expectations of MS-based global studies. In our case, the use of an in-solution digestion protocol with urea buffer and the use of MOAC for proteomics and phosphoproteomics, respectively, would have resulted in a disappointing quantitative analysis for a MS-global study on precious AML patient samples. We therefore encourage some methodology testing before the preparation of samples from cohorts of cancer patients.

Although the classical in-solution digestion protocol with urea lysis buffer has provided satisfactory phosphoproteomic results in previous AML studies [11,12,13], our comparison study showed that it did not perform as well as the in-solution digestion protocol with guanidinium hydrochloride lysis buffer and FASP. A previous comparison of different lysis buffers used in the in-solution digestion method also showed a poor protocol performance when using the urea lysis buffer [34]. The use of the in-solution method with guanidinium hydrochloride, which simultaneously performs reduction and alkylation at high temperature and therefore preserves the sample integrity and its phosphorylation status, resulted in a high number of quantified proteins. However, a high percentage of missed cleavages in the peptide sequences and a poor reproducibility of the phospho-enrichment workflows using peptide samples obtained with this method discouraged us from its further use at a large scale [15].

We realize that FASP might appear as a complicated procedure when compared to in-solution methods that require fewer steps to produce peptides for MS analysis. The sample loss that might occur when working with unknown faulty filters discourages the use of this method despite its high quantification efficiency as has recently been described in depth [25]. However, we have shown that a simple quality test of the spinning filters easily selects the filters that will work properly.

Classical proteomic workflows are still very much on use for the study of clinical samples. For example, in-solution digestion workflows have recently been applied to the study of other cancer samples such as those from colorectal tumors [35] and from body fluids of head and neck cancer patients [36]. Lately, 2DE has been used to analyze saliva from individuals with and without oral leukoplakia [37]. However, as found in our study on AML patient samples, the FASP procedure and the use of a super-SILAC mix of five cell lines [38] have proved to be excellent methodologies for quantitative proteomics of breast cancer samples [39].

From our phospho-enrichment tests, we noticed a poor performance of the MOAC methodology which was reflected in an unsatisfactory performance of SIMAC. However, IMAC produced a high number of phosphopeptides that contained one or several phosphate groups.

To conclude, we have shown the importance of carrying out an initial testing on different sample preparation methods before the analysis of large patient cohorts. We have demonstrated that the FASP procedure followed by MM fractionation and IMAC phospho-enrichment are excellent workflows for the proteome and phosphoproteome analysis, respectively, of AML patient samples by MS. In fact, we have recently identified 8702 SILAC-labeled proteins and 16,346 phosphosites with 20 µg and 600 µg (on average) of AML sample, respectively, using our selected FASP-based workflows in an AML patient cohort (unpublished data, [40]). Therefore, we encourage the use of the FASP technique coupled to a StageTip fractionating step and IMAC phospho-enrichment as excellent alternatives to classical procedures for the study of the AML proteome and phosphoproteome. The use of the FASP strategies can be further applied to the study of secretomes and other PTM.

## Figures and Tables

**Table 1:** Quantitative analysis of different proteomic workflows ^1^.

Method ^2^ (SILAC Labeling)	Quantified Protein Groups	Quantified Peptides	Missed Cleavages ^3^ (%)
In-solution digestion, U, SD	1011	8082	42
In-solution digestion, U, SD, MM	1627	11,096	29
In-solution digestion, U, SD, SCX	1201	7948	29
In-solution digestion, G, DD	1091	10,816	55
In-solution digestion, G, DD, MM	2006	15,894	31
In-solution digestion, G, DD, SCX	2051	16,110	35
FASP, SD	1480	12,607	15
FASP, SD, MM	2141	16,469	11
FASP, SD, SCX	1500	10,414	11

^1^ Data obtained from our testing study on proteomic workflows using SILAC-labeled AML patient samples [15]. Two technical replicates were used in the workflow test; ^2^ SD and DD stand for single and double digestion, respectively, U stands for urea, G stands for guanidinium hydrochloride, MM stands for mixed mode, SCX stands for strong cation exchange, FASP stands for filter-aided sample preparation; ^3^ The percentage of missed cleavages is calculated taken into account peptides with one or more missed cleavages.

**Table 2:** Quantitative analysis of different FASP-based phospho-enrichment techniques ^1^.

Method ^2^ (SILAC Labeling)	Localized Phosphosites ^3^	Mono-Phosphopeptides	Di- and Multi-Phosphopeptides
IMAC, SD	2708	2745	607
IMAC, DD	2792	2489	843
MOAC, SD	738	945	29
MOAC, DD	897	1118	46
SIMAC, SD	1817	883	954
SIMAC, DD	1825	998	883

^1^ Data obtained from our testing study on phosphoproteomic methodologies using SILAC-labeled FASP-prepared peptides from AML patient samples [15]. The number of phosphopeptides and phosphosites represents the average of two biological replicates; ^2^ SD and DD stand for single and double digestion, respectively; ^3^ Localization probability of at least 75%.

## Abbreviations

1DE	One-dimensional electrophoresis
2DE	Two-dimensional electrophoresis
AAI	Anti-apoptosis index
AML	Acute myeloid leukemia
CAA	Chloroacetamide
CID	Collision-induced dissociation
DDA	Data-dependent acquisition
ESI	Electrospray ionization
FAB	French-American-British
FASP	Filter-aided sample preparation
GASP	Gel-aided sample preparation
IMAC	Immobilized metal affinity chromatography
KSEA	Kinase-substrate enrichment analysis
LC	Liquid chromatography
LDS	Lithium dodecyl sulfate
MALDI-TOF	Matrix-assisted laser desorption/ionization time-of-flight
MM	Mixed mode
MOAC	Metal oxide affinity chromatography
MS	Mass spectrometry
PTM	Post-translational modifications
SCX	Strong cation exchange
SDB-RPS	Polystyrenedivinylbenzene reversed phase sulfonate
SDS	Sodium dodecyl sulfate
SH2	Src homology 2
SILAC	Stable isotope labelling with amino acids in cell culture
SIMAC	Sequential elution from IMAC
TCEP	Tris(2-carboxyethyl)phosphine
WHO	World Health Organization

## References

[1] Estey E.H. (2014). Acute myeloid leukemia: 2014 Update on risk-stratification and management. Am. J. Hematol..

[2] Lowenberg B., Downing J.R., Burnett A. (1999). Acute myeloid leukemia. N. Engl. J. Med..

[3] Howlader N., Noone A.M., Krapcho M., Miller D., Bishop K., Altekruse S.F., Kosary C.L., Yu M., Ruhl J., Tatalovich Z. Leukemia: SEER Cancer Statistics Review 1975–2013. http://seer.cancer.gov/csr/1975_2013/.

[4] Bennett J.M., Catovsky D., Daniel M.T., Flandrin G., Galton D.A., Gralnick H.R., Sultan C. (1985). Proposed revised criteria for the classification of acute myeloid leukemia: A report of the French-American-British Cooperative Group. Ann. Intern. Med..

[5] Vardiman J.W., Thiele J., Arber D.A., Brunning R.D., Borowitz M.J., Porwit A., Harris N.L., Le Beau M.M., Hellstrom-Lindberg E., Tefferi A. (2009). The 2008 revision of the world health organization (who) classification of myeloid neoplasms and acute leukemia: Rationale and important changes. Blood.

[6] Godley L.A. (2014). Inherited predisposition to acute myeloid leukemia. Semin. Hematol..

[7] Dohner H., Weisdorf D.J., Bloomfield C.D. (2015). Acute myeloid leukemia. N. Engl. J. Med..

[8] Roboz J., Roboz G.J. (2015). Mass spectrometry in leukemia research and treatment. Expert Rev. Hematol..

[9] Aasebo E., Forthun R.B., Berven F., Selheim F., Hernandez-Valladares M. (2016). Global cell proteome profiling, phospho-signaling and quantitative proteomics for identification of new biomarkers in acute myeloid leukemia patients. Curr. Pharm. Biotechnol..

[10] Luczak M., Kazmierczak M., Handschuh L., Lewandowski K., Komarnicki M., Figlerowicz M. (2012). Comparative proteome analysis of acute myeloid leukemia with and without maturation. J. Proteom..

[11] Casado P., Rodriguez-Prados J.C., Cosulich S.C., Guichard S., Vanhaesebroeck B., Joel S., Cutillas P.R. (2013). Kinase-substrate enrichment analysis provides insights into the heterogeneity of signaling pathway activation in leukemia cells. Sci. Signal..

[12] Schaab C., Oppermann F.S., Klammer M., Pfeifer H., Tebbe A., Oellerich T., Krauter J., Levis M., Perl A.E., Daub H. (2014). Global phosphoproteome analysis of human bone marrow reveals predictive phosphorylation markers for the treatment of acute myeloid leukemia with quizartinib. Leukemia.

[13] Jin L.L., Wybenga-Groot L.E., Tong J., Taylor P., Minden M.D., Trudel S., McGlade C.J., Moran M.F. (2015). Tyrosine phosphorylation of the Lyn Src homology 2 (SH2) domain modulates its binding affinity and specificity. Mol. Cell. Proteom..

[14] Wojtuszkiewicz A., Schuurhuis G.J., Kessler F.L., Piersma S.R., Knol J.C., Pham T.V., Jansen G., Musters R.J., van Meerloo J., Assaraf Y.G. (2016). Exosomes secreted by apoptosis-resistant acute myeloid leukemia (aml) blasts harbor regulatory network proteins potentially involved in antagonism of apoptosis. Mol. Cell. Proteom..

[15] Aasebo E., Mjaavatten O., Vaudel M., Farag Y., Selheim F., Berven F., Bruserud O., Hernandez-Valladares M. (2016). Freezing effects on the acute myeloid leukemia cell proteome and phosphoproteome revealed using optimal quantitative workflows. J. Proteom..

[16] Wisniewski J.R., Zougman A., Nagaraj N., Mann M. (2009). Universal sample preparation method for proteome analysis. Nat. Methods.

[17] Thingholm T.E., Larsen M.R. (2016). Phosphopeptide enrichment by immobilized metal affinity chromatography. Methods Mol. Biol..

[18] Thingholm T.E., Larsen M.R. (2016). The use of titanium dioxide for selective enrichment of phosphorylated peptides. Methods Mol. Biol..

[19] Thingholm T.E., Larsen M.R. (2016). Sequential elution from *IMAC* (SIMAC): An efficient method for enrichment and separation of mono- and multi-phosphorylated peptides. Methods Mol. Biol..

[20] Montoya A., Beltran L., Casado P., Rodriguez-Prados J.C., Cutillas P.R. (2011). Characterization of a TiO_2_ enrichment method for label-free quantitative phosphoproteomics. Methods.

[21] St-Germain J.R., Taylor P., Tong J., Jin L.L., Nikolic A., Stewart I.I., Ewing R.M., Dharsee M., Li Z., Trudel S. (2009). Multiple myeloma phosphotyrosine proteomic profile associated with FGFR3 expression, ligand activation, and drug inhibition. Proc. Natl. Acad. Sci. USA.

[22] Manza L.L., Stamer S.L., Ham A.J., Codreanu S.G., Liebler D.C. (2005). Sample preparation and digestion for proteomic analyses using spin filters. Proteomics.

[23] Wisniewski J.R., Mann M. (2012). Consecutive proteolytic digestion in an enzyme reactor increases depth of proteomic and phosphoproteomic analysis. Anal. Chem..

[24] Wisniewski J.R., Rakus D. (2014). Multi-enzyme digestion fasp and the ‘total protein approach’-based absolute quantification of the *Escherichia coli* proteome. J. Proteom..

[25] Wisniewski J.R. (2016). Quantitative evaluation of filter aided sample preparation (FASP) and multienzyme digestion FASP protocols. Anal. Chem..

[26] Kulak N.A., Pichler G., Paron I., Nagaraj N., Mann M. (2014). Minimal, encapsulated proteomic-sample processing applied to copy-number estimation in *Eukaryotic* cells. Nat. Methods.

[27] Scheltema R.A., Hauschild J.P., Lange O., Hornburg D., Denisov E., Damoc E., Kuehn A., Makarov A., Mann M. (2014). The Q exactive HF, a Benchtop mass spectrometer with a pre-filter, high-performance quadrupole and an ultra-high-field Orbitrap analyzer. Mol. Cell. Proteom..

[28] Kelstrup C.D., Jersie-Christensen R.R., Batth T.S., Arrey T.N., Kuehn A., Kellmann M., Olsen J.V. (2014). Rapid and deep proteomes by faster sequencing on a benchtop quadrupole ultra-high-field Orbitrap mass spectrometer. J. Proteome Res..

[29] Fischer R., Kessler B.M. (2015). Gel-aided sample preparation (GASP)—A simplified method for gel-assisted proteomic sample generation from protein extracts and intact cells. Proteomics.

[30] Aasebo E., Vaudel M., Mjaavatten O., Gausdal G., Van der Burgh A., Gjertsen B.T., Doskeland S.O., Bruserud O., Berven F.S., Selheim F. (2014). Performance of super-silac based quantitative proteomics for comparison of different acute myeloid leukemia (AML) cell lines. Proteomics.

[31] Siepen J.A., Keevil E.J., Knight D., Hubbard S.J. (2007). Prediction of missed cleavage sites in tryptic peptides aids protein identification in proteomics. J. Proteome Res..

[32] Hernandez-Valladares M., Aasebo E., Mjaavatten O., Vaudel M., Bruserud O., Berven F., Selheim F. (2016). Reliable FASP-based procedures for optimal quantitative proteomic and phosphoproteomic analysis on samples from acute myeloid leukemia patients. Biol. Proced. Online.

[33] Fila J., Honys D. (2012). Enrichment techniques employed in phosphoproteomics. Amino Acids.

[34] Leon I.R., Schwammle V., Jensen O.N., Sprenger R.R. (2013). Quantitative assessment of in-solution digestion efficiency identifies optimal protocols for unbiased protein analysis. Mol. Cell. Proteom..

[35] Li M., Peng F., Li G., Fu Y., Huang Y., Chen Z., Chen Y. (2016). Proteomic analysis of stromal proteins in different stages of colorectal cancer establishes Tenascin-C as a stromal biomarker for colorectal cancer metastasis. Oncotarget.

[36] Jehmlich N., Stegmaier P., Golatowski C., Salazar M.G., Rischke C., Henke M., Volker U. (2016). Proteome data of whole saliva which are associated with development of oral mucositis in head and neck cancer patients undergoing radiotherapy. Data Brief.

[37] Camisasca D.R., da Ros Goncalves L., Soares M.R., Sandim V., Nogueira F.C., Garcia C.H., Santana R., de Oliveira S.P., Buexm L.A., de Faria P.A. (2016). A proteomic approach to compare saliva from individuals with and without oral leukoplakia. J. Proteom..

[38] Geiger T., Cox J., Ostasiewicz P., Wisniewski J.R., Mann M. (2010). Super-silac mix for quantitative proteomics of human tumor tissue. Nat. Methods.

[39] Pozniak Y., Balint-Lahat N., Rudolph J.D., Lindskog C., Katzir R., Avivi C., Ponten F., Ruppin E., Barshack I., Geiger T. (2016). System-wide clinical proteomics of breast cancer reveals global remodeling of tissue homeostasis. Cell Syst..

[40] Hernandez-Valladares M., Aasebø E., Vaudel M., Berven F.S., Selheim F., Doskeland S.Ø., Bruserud Ø. (2016).

